# Management of initial colonisations with *Burkholderia* species in France, with retrospective analysis in five cystic fibrosis Centres: a pilot study

**DOI:** 10.1186/s12890-020-01190-y

**Published:** 2020-06-05

**Authors:** Vianney Gruzelle, Hélène Guet-Revillet, Christine Segonds, Stéphanie Bui, Julie Macey, Raphaël Chiron, Marine Michelet, Marlène Murris-Espin, Marie Mittaine

**Affiliations:** 1 CRCM pédiatrique, Service de Pneumo-Allergologie pédiatrique, Hôpital des Enfants, Centre Hospitalier Universitaire de Toulouse, 330 avenue de Grande-Bretagne - TSA 40031, 31059 Toulouse cedex 9, France; 2 Service de Bactériologie-Hygiène, Centre Hospitalier Universitaire de Toulouse, Toulouse, France; 3 Observatoire Burkholderia cepacia, Centre Hospitalier Universitaire de Toulouse, Toulouse, France / Vaincre la Mucoviscidose, Paris, France; 4 CRCM pédiatrique, Service de pédiatrie médicale, Centre Hospitalier Universitaire de Bordeaux-GH Pellegrin, Bordeaux, France; 5 Service de pneumologie, Centre Hospitalier Universitaire de Bordeaux-GH Sud - Hôpital Haut-Lévêque, Pessac, France; 6 CRCM pédiatrique, Service des maladies respiratoires, Centre Hospitalier Universitaire de Montpellier - Hôpital Arnaud de Villeneuve, Montpellier, France; 7 Service de pneumologie - Consultation Mucoviscidose, Pôle voies respiratoires, Centre Hospitalier Universitaire de Toulouse - Hôpital Larrey, Toulouse, France

**Keywords:** *Burkholderia cepacia* complex, BCC, *Burkholderia gladioli*, Cystic fibrosis, Eradication

## Abstract

**Background:**

Whereas *Burkholderia* infections are recognized to impair prognosis in cystic fibrosis (CF) patients, there is no recommendation to date for early eradication therapy. The aim of our study was to analyse the current management of initial colonisations with *Burkholderia cepacia* complex (BCC) or *B. gladioli* in French CF Centres and its impact on bacterial clearance and clinical outcome.

**Methods:**

We performed a retrospective review of the primary colonisations (PC), defined as newly positive sputum cultures, observed between 2010 and 2018 in five CF Centres. Treatment regimens, microbiological and clinical data were collected.

**Results:**

Seventeen patients (14 with BCC, and 3 with *B. gladioli*) were included. Eradication therapy, using heterogeneous combinations of intravenous, oral or nebulised antibiotics, was attempted in 11 patients. Six out of the 11 treated patients, and 4 out of the 6 untreated patients cleared the bacterium. Though not statistically significant, higher forced expiratory volume in 1 second and forced vital capacity at PC and consistency of treatment with in vitro antibiotic susceptibility tended to be associated with eradication. The management of PC was shown to be heterogeneous, thus impairing the statistical power of our study. Large prospective studies are needed to define whom to treat, when, and how.

**Conclusions:**

Pending these studies, we propose, due to possible spontaneous clearance, to check the presence of *Burkholderia* 1 month after PC before starting antibiotics, at least in the milder cases, and to evaluate a combination of intravenous beta-lactam + oral or intravenous fluoroquinolone + inhaled aminoglycoside.

## Background

The *Burkholderia cepacia* complex (BCC) is a group of 22 closely related species belonging to the *Burkholderia* genus, which are capable of colonising and infecting the respiratory epithelium of cystic fibrosis (CF) patients. Though asymptomatic carriage is possible, BCC organisms are associated with a marked increase in morbidity-mortality rates, due to rapid decline in lung function, extensive pneumopathy with life-threatening septicaemia (cepacia syndrome), as well as to poor lung transplantation outcomes [1–3]. *B. cenocepacia* and *B. multivorans* are the main species involved in North America and most European countries including France [3–6], whereas a high prevalence of *B. contaminans* is observed in Ibero-American countries [7]. In the Iberian Peninsula*, B. cepacia* and *B. stabilis* are more frequent than in other European countries [8, 9]. Several studies demonstrated faster clinical deterioration, as well as lower survival after transplantation in patients harbouring *B. cenocepacia* compared to those harbouring *B. multivorans* [2, 3, 10, 11]. However, the spread of the ST180 strain of *B. multivorans* in a French CF Centre resulted in numerous cepacia syndromes and deaths (French Observatoire *Burkholderia cepacia* (OBC) data). The impact of other species of the BCC is less documented, though adverse outcomes have also been reported [7, 12]. In the 1990s, highly transmissible and often highly virulent strains were responsible for large outbreaks within CF communities [13–15]. Following the implementation of infection control measures, patient-to-patient transmission decreased, and the distribution of BCC species was hence modified. Thus, in countries where *B. cenocepacia* was the most common species in the 1990s due to epidemic spread, *B. multivorans* currently outweighs *B. cenocepacia* in new infections [3, 5, 6]. The impact of *B. gladioli* colonisation is less clear, but abscesses and bacteraemia following lung transplantation have been reported [16, 17]. In Europe and the United States, the current prevalence and incidence of BCC infections are about 2 and 0.5%, respectively, whereas the prevalence of *B. gladioli* is below 0.5% [4, 6].

Early eradication therapy of *Pseudomonas aeruginosa* has been clearly proven to be a successful strategy to postpone chronic infection. Conversely, studies evaluating the treatment of newly acquired BCC colonisations, given their low incidence, are sparse and based on isolated case reports or small series, which are often inconclusive [18]. There are no recommendations either concerning the even rarer *B. gladioli*, though it is one of the most troublesome species during the peri-transplantation period. Moreover, *Burkholderia* species are multi-drug resistant, which limits therapeutic options, and in vitro susceptibility testing is considered to be poorly reliable [19].

In this context, the aim of this preliminary study was to evaluate the current approach to new infections with *Burkholderia* in France, and the impact of potential eradication treatment on bacterial clearance and pulmonary outcomes. We present a 2010–2018 retrospective study in five CF Centres, including 17 patients.

## Methods

### Ethics statement

The data collection process was reported to the Commission Nationale Informatique et Liberté (French Data Protection Authority) on 26/01/2018, under number 2146344v0.

### Patient recruitment and data collection

Five French CF Centres (3 paediatric and 2 adult Centres), located in Toulouse, Montpellier and Bordeaux, as well as the French OBC participated in the present pilot study. In all Centres, the microbiological analysis of sputum samples was performed using a selective medium and a low detection level for *Burkholderia* species, according to the guidelines of the French Society for Microbiology [20]. Patients who acquired BCC or *B. gladioli* between 01/01/2010 and 01/01/2018 were considered for retrospective analysis. The absence of *Burkholderia* from all sputum cultures during the two prior years was required for inclusion. The date of primary colonisation (PC) was defined as the date of the first positive sputum culture, and the primary isolate had to have been sent to the OBC for analysis. Patient data were collected retrospectively from computerised regional registers and medical records in each CF Centre. Baseline characteristics included gender, age, CFTR (Cystic Fibrosis Transmembrane conductance Regulator) genotype, body mass index (BMI), best forced vital capacity (FVC) and best forced expiratory volume in 1 second (FEV1), as well as exocrine pancreatic insufficiency, insulin-dependent diabetes, nutritional support, liver cirrhosis, allergic broncho-pulmonary aspergillosis (ABPA), and need for systemic antibiotic treatment or corticotherapy in the 3 months preceding PC. To assess lung function evolution, the FEV1 values 2 years after PC were recorded.

Microbiological data included the co-pathogens at PC (*Pseudomonas aeruginosa*, methicillin-susceptible and methicillin-resistant *Staphylococcus aureus*, *Achromobacter* spp., *Stenotrophomonas maltophilia*, Fungi and Mycobacteria), the number of sputum cultures performed during the 2 years preceding and the 2 years following PC, as well as the dates of the last positive and of the first negative cultures. The *Burkholderia* strains that were recovered only once (from the PC sputum), and never during the follow-up period, were defined as single isolates.

When eradication therapy directed against *Burkholderia* was implemented, onset date, molecules used, routes of administration, and duration were recorded.

### Strain analysis

Identification of the primary and potential subsequent isolates to the species-level was determined by the OBC, using ARDRA (Amplified Ribosomal DNA-Restriction Analysis) [17, 21], or *RecA* gene sequencing [22]. Strain analysis of BCC isolates was complemented by genotyping to assess BCC cross-transmission if relevant, i.e. if the same species had been recovered from at least one other patient in the same CF Centre within the 3 preceding years. Besides, all *B. cenocepacia* and *B. multivorans* isolates were compared to the highly transmissible strains identified in France.

Once data collection had been completed, the susceptibility of the last strain recovered before the onset of treatment was tested by the OBC, for each antibiotic administered, following the guidelines of the antibiogram committee of the French Society for Microbiology, by means of the disk diffusion test (piperacillin-tazobactam, cotrimoxazole), or the epsilometer test (ceftazidime, temocillin, aztreonam, levofloxacin, ciprofloxacin). Results were interpreted using PK-PD breakpoints, or species related breakpoints when available [23]. In the absence of specific values for inhaled aztreonam-lysine, the breakpoints for parenteral administration were used, whereas inhaled tobramycin was considered active given the high pulmonary concentrations delivered [24, 25]. The consistency of eradication treatments with in vitro antibiotic susceptibilities or recognized activity (inhaled tobramycin) was then analysed for each patient treated. We considered the treatment as matching the antibiogram when the bacterial strain was fully susceptible to at least two antibiotics, or to at least one antibiotic if combined with inhaled tobramycin, because of the high expected local tobramycin concentrations.

### Microbiological outcome

The microbiological follow-up allowed the classification of patients into two groups: “eradication”, when the bacterium was not recovered over a follow-up period of at least 1 year including ≥3 sputum cultures, and “persistence” in case of chronic colonisation. In the “persistence” group, the in vitro antibiotic susceptibility of the post-treatment isolate was compared to that of the pre-treatment isolate.

### Statistical analysis

After a verification phase in order to highlight missing, incorrect or inconsistent data, the database was locked, and information analysed. Characteristics were compared via the Chi2 test or Fisher’s exact test using theoretical aspects for qualitative variables. Comparisons of quantitative variables were performed using the Student test, or the Mann-Whitney non-parametric test if the normality or homoscedasticity of the distributions had not been checked. Statistical tests were conducted with STATA v13.1, and were carried out using a bilateral approach and a type one error of 5%. A value of *p* < 0.05 was considered significant for all analyses.

## Results

### General characteristics

The general characteristics of the cohort are described in Table 1. Seventeen patients (7 children < 18 years and 10 adults ≥18 years) were included. At least four sputum cultures had been performed during the two previous years and were *Burkholderia* negative. The sex ratio (M/F) was 1/0.3. Most patients (15/17) carried the F508del mutation, with a majority of heterozygotes (12/15). Median FEV1 at PC was 83% predicted and 7 patients had a FEV1 ≥ 90%. During the 3 months preceding PC, 70.6% (12/17) of patients had received antibiotic therapy due to clinical condition’s deterioration, and 17.6% (3/17), systemic corticosteroids. Fourteen patients were colonised with BCC species, the most common species being *B. multivorans* (6 patients). Bacterial genotyping demonstrated that all BCC PC involved sporadic strains, with the exception of one epidemic strain of *B. multivorans*. Three patients were colonised with *B. gladioli*. A majority of patients (12/17, 70.6%) also harboured *S. aureus,* and 6/17 (35.3%) *P. aeruginosa*.

**Table 1:** Baseline characteristics of the 17 patients at *Burkholderia* primary colonisation

Patient characteristics (***N*** = 17)	Number (percentage) or median value [min-max]
Male gender	13 (76.5%)
Age (years)	19 [6–34]
CFTR genotype: F508del/F508del	3 (17.6%)
F508del/other or unknown	12 (70.6%)
Other or unknown	2 (11.8%)
BMI (Z-score)	−0.76 [−2.81–0.39]
Insulin-dependent diabetes	3 (17.6%)
Exocrine pancreatic insufficiency	16 (94.1%)
Nutritional support (gastrostomy/enteral tube)	1 (5.9%)
Liver cirrhosis	1 (5.9%)
ABPA	3 (17.6%)
**Respiratory function tests**	
Median FEV1 (% predicted)	83.0 [26–113]
Median FVC (% predicted)	92.0 [36–109]
***Burkholderia*species**	
**BCC**	**14 (82.4%)**
*B. multivorans*	6 (35.3%)
*B. cenocepacia*^*a*^	4 (23.5%)
*B. contaminans*	1 (5.9%)
*B. vietnamiensis*	1 (5.9%)
*B. ambifaria*	1 (5.9%)
Unidentified BCC^b^	1 (5.9%)
***B. gladioli***	**3 (17.6%)**
**Co-pathogens**	
Methi-S *Staphylococcus aureus*	7 (41.2%)
Methi-R *Staphylococcus aureus*	5 (29.4%)
*Pseudomonas aeruginosa*	6 (35.3%)
Fungi	4 (23.5%)
*Stenotrophomonas maltophilia*	3 (17.6%)
Non-tuberculous Mycobacteria	2 (11.8%)
*Achromobacter* spp*.*	0 (0%)

*CFTR* Cystic Fibrosis Transmembrane conductance Regulator, *BMI* Body Mass Index, *ABPA* Allergic Broncho-Pulmonary Aspergillosis, *FEV1* Forced Expiratory Volume in one second, *FVC* Forced Vital Capacity, *BCC Burkholderia cepacia* complex, *Methi-S* Methicillin**-**Susceptible**,***Methi-R* Methicillin-Resistant

^a^ including 3 *cenocepacia* IIIA and 1 *cenocepacia* IIIB subtypes

^b^on the basis of *RecA* sequencing results analysed using the *Burkholderia cepacia* complex MLST Databases website (https://pubmlst.org/bcc/), this isolate was confirmed as BCC, but could not be assigned to any of the BCC species described so far

### Eradication treatment modalities

PC treatments and outcomes are detailed per patient in Table 2. Eleven out of 17 patients (64.7%) underwent eradication therapy, including 4 out of 6 patients with *B. multivorans*, 4 out of 4 patients with *B. cenocepacia*, 2 out of 4 patients with other BCC species, and 1 out of 3 patients with *B. gladioli*. The median interval between PC and the onset of treatment was 38 days [range 5–98]. Treatment modalities were heterogeneous, including intravenous (IV), oral and nebulised antibiotics. All antibiotic regimens were combinations of two compounds or more and comprised an IV beta-lactam in 8 cases (72.7%), combined with an IV (4) or inhaled (2) aminoglycoside and/or IV ciprofloxacin (3). Oral treatments (4 patients) included levofloxacin or ciprofloxacin and/or cotrimoxazole. Three patients received inhaled aztreonam-lysine. The duration of all IV courses was 14 days, whereas that of oral treatments was 21 to 28 days. Lastly, the treatment matched antibiogram data in 54.5% of patients (6/11).

**Table 2:** Individual characteristics, treatments and outcomes in 17 cases of *Burkholderia* primary colonisation

Patient No.	Centre	*Burkholderia* Species	Co-pathogens	Age (years)	FEV1% at PC	Treatment (molecules, routes of administration and duration)	Consistency antibiogram/ antibiotic therapy^a^	Outcome
1	M	*B. multivorans*	MRSA, PA	19	71	PO Levofloxacin and Cotrimoxazole 21 days; INH Aztreonam 28 days	Yes (3/3)	Eradication
2	M	*B. multivorans*	MRSA, PA, Fungi^e^	23	79	PO Cotrimoxazole; INH Aztreonam, 28 days	Yes (2/2)	Eradication
3	M	*B. multivorans*	MSSA, Fungi^e^	28	94	IV Ceftazidime and Tobramycin, 14 days	No (1/2)	Persistence
4^b^	B	*B. multivorans*	MRSA	13	31	IV Ceftazidime and Gentamicin, 14 days	No (1/2)	Persistence
5	T	*B. multivorans*		6	113	Not treated		Eradication^c^
6	T	*B. multivorans*	MSSA, PA, Fungi^e^	21	105	Not treated		Persistence
7	T, B	*B. cenocepacia* IIIA	MRSA, *Sten*	10	87	IV Ceftazidime and Ciprofloxacin, 14 days	Yes (2/2)	Eradication^c^
8	M	*B. cenocepacia* IIIB	*Sten*, MB	18	52	PO Ciprofloxacin; INH Aztreonam, 28 days	No (0/2)	Eradication^c^
9	T	*B. cenocepacia* IIIA	MRSA PA	19	59	IV Piperacillin-tazobactam and Ciprofloxacin, 14 days	No (1/2)^*d*^	Persistence
10	M	*B. cenocepacia* IIIA	PA	22	62	IV Temocillin and Tobramycin, 14 days; PO Cotrimoxazole, 28 days	Yes (2/3)	Persistence
11	M	*B. contaminans*	PA, MB	33	26	Not treated		Eradication^c^
12	T, M	*B. vietnamiensis*	MSSA	17	109	Not treated		Eradication
13	B	*B. ambifaria*	MSSA	7	83	IV Ceftazidime and Tobramycin, 14 days	No (1/2)	Persistence
14	T	Unidentified BCC	MSSA	16	106	IV Ceftazidime and Ciprofloxacin; INH Tobramycin, 14 days	Yes (3/3)	Eradication^c^
15	B	*B. gladioli*	MSSA	14	112	IV Piperacillin-tazobactam; INH Tobramycin, 14 days	Yes (2/2)	Eradication
16	T	*B. gladioli*	*Sten*	34	99	Not treated		Eradication
17	T	*B. gladioli*	MSSA, Fungi^e^	32	65	Not treated		Persistence

*FEV1* Forced Expiratory Volume in one second, *B* Bordeaux, *M* Montpellier, *T* Toulouse, *BCC Burkholderia cepacia* complex, *MB* Mycobacteria, *MSSA*, Methicillin-Susceptible *Staphylococcus aureus*, *MRSA* Methicillin-Resistant *Staphylococcus aureus, PA Pseudomonas aeruginosa*, *Sten Stenotrophomonas maltophilia, PO* per os*, IV* intravenous, *INH* inhaled

^a^ Consistency antibiogram/antibiotic therapy: in vitro full susceptibility to at least two of the antibiotics used, or to at least one antibiotic if combined with inhaled tobramycin. All BCC were resistant to IV aminoglycosides (natural resistance)

^b^ This patient underwent lung transplantation and colonisation disappeared following surgery

^c^ Single isolate

^*d*^ The strain was only susceptible to ciprofloxacin

^e^ Concerning fungi repartition: patient No 2 *Aspergillus fumigatus*; Patient No 3 *Aspergillus spp*.; Patient No 6 *Candida albicans*; Patient No 17 *Aspergillus fumigatus* and *Candida albicans*

### Outcomes

The median duration of follow-up was 5.9 years [range 1.0–8.2]. There was no death, *Burkholderia*-induced septicaemia, nor cepacia syndrome to report. One patient underwent lung transplantation 2 years after PC. The *Burkholderia* strain was cleared in 10 patients, whereas infection was persistent in 7 patients. A comparison of patient characteristics in the two microbiological outcome groups is presented in Table 3. There was no significant difference in terms of gender, age, previous antibiotherapy, BMI, and bacterial species. FEV1 values at PC and 2 years after PC were lower in the “persistence” group than in the “eradication” group, but the difference did not reach statistical significance (*p* = 0.12 and *p* = 0.08, respectively). No correlation was found between implementation of eradication therapy, its time of onset and microbiological outcome. Four of the 6 untreated patients spontaneously cleared the bacterium, which was isolated only once in 2 of these patients, as well as in 3/6 treated patients. Eradication was obtained in 5/6 patients receiving an antibiotic regimen which was in accordance with antibiogram data, and in only 1/5 patients otherwise (*p* = 0.08). It should be noted that beta-lactam/IV aminoglycosides combinations (+/− another molecule) were associated with treatment failures in 4/4 patients, even when the strain was susceptible to the beta-lactam used, whereas the two eradication regimens including inhaled tobramycin were successful. Five patients received combinations including a fluoroquinolone (ciprofloxacin or levofloxacin), which was active in vitro in 3 cases belonging to the “eradication” group (with 1/3 single isolation), and inactive in 2 cases (1 in the “eradication” group with a single isolation, and 1 in the “persistence” group). Lastly, inhaled aztreonam-lysine was associated with eradication in 3/3 patients, with 2/3 single isolations. Concerning the impact of treatment on antibiotic susceptibility, the analysis of post-treatment isolates in the 5 treated patients who developed chronic infection showed that 2/5 isolates had become resistant to one of the antibiotics administered (1 to ciprofloxacin and 1 to cotrimoxazole), whereas beta-lactam susceptibilities were not significantly modified.

**Table 3:** Compared patient data in the “eradication” and “persistence” groups. Results are expressed in numbers or mean ± standard deviation

Patient data	Eradication (***N*** = 10)	Persistence (***N*** = 7)	***p*** value
Male	8	5	1
Age at PC (years)	19.1 ± 8.6	20.2 ± 8.0	0.74
BMI (Z-score) at PC	−1.04 ± 0.8	−0.62 ± 0.6	0.42
Antibiotics in the 3 months before PC	9	3	0.1
**Lung function (% predicted)**			
FVC at PC	92.8 ± 15.6	65.3 ± 20.1	0.11
FEV1 at PC	85.4 ± 27.4	65.7 ± 23.0	0.12
FEV1 2 years after PC^a^	77.7 ± 26.1	57.2 ± 18.4	0.08
**Distribution of*Burkholderia*strains at PC**			
**BCC**	8	6	
*B. multivorans*	3	3	0.64
*B. cenocepacia*	2	2	1
*B. contaminans*	1	0	1
*B. vietnamiensis*	1	0	1
*B. ambifaria*	0	1	0.41
Unidentified BCC	1	0	1
***B. gladioli***	2	1	1
**Antibiotic treatment**			
No treatment	4	2	1
Eradication treatment	6	5	1
Time to treat from the PC (days)	34 ± 20.5	48 ± 37.9	0.71
**Consistency antibiogram/antibiotic therapy**			
Consistent^b^	5	1	0.08
Inconsistent	1	4	

*BMI* Body Mass Index, *PC* primary colonisation, *FVC* Forced Vital Capacity*, FEV1* Forced Expiratory Volume in one second, *BCC Burkholderia cepacia* complex

^a^ Data unavailable for one patient with one-year follow-up,

^b^ in vitro full susceptibility to at least two of the antibiotics used, or to at least one antibiotic if combined with inhaled tobramycin

## Discussion

Though limited to 5 of the 40 French CF Centres following *Burkholderia*-positive patients, our retrospective study demonstrated a great diversity in the management of initial colonisations with BCC or *B. gladioli*.

### Should eradication therapy be systematically implemented?

In the present series, eradication was not systematically attempted, as related by Horsley et al. in their survey of adult CF Centres in UK [26]. Indeed, while in the epidemic era, most infections with BCC persisted over years, nowadays infection is transient in about 50% of French patients [6]. As regards *B. gladioli*, Kennedy et al. showed a transient colonisation rate of 60% in a series of 30 North American patients [16], similar to that observed in France [17]. In the present study, spontaneous clearance was observed in 4 out of 6 untreated patients, and in the 3 treated patients with only one positive sputum culture, we cannot be sure that the bacterium would not have been cleared without antibiotic therapy, as pointed out by Horsley et al. in their study [26]. These observations could lead to discuss a short follow-up of sputum cultures before introducing eradication therapy in some patients whose characteristics have yet to be defined in greater detail.

Some risk factors for progressing towards chronic colonisation have been proposed. In a study including 64 patients in Australia, Ramsay et al. reported a significantly higher risk of chronic infection in adults than in children [27]. However, in our small cohort, patients in the «persistence» group were not significantly older than those in the “eradication” group. Folescu et al. studied BCC infections in 27 Brazilian patients chronically colonised with *P. aeruginosa* and pointed out a correlation between a low BMI and chronic colonisation [28]. The major difference, though not statistically significant, between our two groups was a lower FEV1 value in case of “persistence”, which agrees with the poorer lung function at PC observed in adult patients with chronic infection by Ramsay et al. [27]. Thus, age and degree of severity of the disease (FEV1 and BMI) at the time of *Burkholderia* acquisition may have an influence on the success of BCC eradication treatment.

### *Burkholderia* species and outcomes

Another question is whether approaches to new infections ought to be adjusted according to the species detected, with regard to the ability to be involved in chronic infection, as well as to potential virulence. In Canada, Zlosnik et al. reported a higher rate of transient infection in patients harbouring *B. multivorans* (55%) or *B. vietnamiensis* (44%), than in patients harbouring *B. cenocepacia* (19%). In the present study, the effect of the *Burkholderia* species on progression to chronic colonisation could not be established given the considerable diversity of the species and the limited number of patients. As regards the clinical impact of infection, the poor outcomes generally associated with *B. cenocepacia* may be partly due to the spread of highly virulent clones in the 1990s, and *B. multivorans*, which is considered to be less virulent than *B. cenocepacia* in most countries, was also responsible for severe infections in French patients (OBC data), [29]. Thus, species-adjusted eradication therapy implementation should take account of national, or even local epidemiology, and defining the attitude to infection with rare species is expected to be difficult. Concerning transplantation-associated risks, *B. cenocepacia* and *B. gladioli* are considered to be the most problematic species and constitute a relative contraindication to lung transplantation. However, we also observed post-transplant bloodstream infections due to *B. multivorans* and *B. vietnamiensis* (OBC data), which does not allow to rule out any risk in case of colonisation with other species. In our study, eradication was attempted in 4 out of 4 patients with *B. cenocepacia*, but in only 1 out of 3 patients with *B. gladioli*, and in 6 out of 10 patients with other species. To-date, there is no evidence clearly establishing the benefit of early eradication therapy [18] and our study failed to demonstrate the superiority of attempted eradication over therapeutic abstention. Nevertheless, as discussed above, it can be assumed that eradication treatment is relevant in patients whose infection is most likely to become chronic, namely those with the most severe forms of the disease at detection, and/or in patients colonised by given species.

### Is in vitro antibiotic susceptibility testing useful to choose an eradication protocol?

Antibiotic regimens were heterogeneous, including IV, oral and nebulised antibiotics. Such heterogeneity was also reported in the UK survey by Horsley et al. [26], and within published studies. Kitt et al. successfully proposed tobramycin, temocillin and ceftazidime IV combination therapy for 14 days followed by a switch to tobramycin aerosols for 3 months in 2 children (1 *B. gladioli* and 1 *B. cepacia*) [30]. Uluer et al. reported 12 failed attempts at eradication of *B. dolosa* following the administration of amiloride and tobramycin aerosols for 6 months [31]. Using the same treatment, Ball et al. recorded 6 therapeutic failures in 7 children treated (eradication of *B. stabilis*, persistence of 3 *B. multivorans* and 3 *B. cenocepacia*) [32]. Among 14 treated patients, Horsley et al. reported 4 successful eradications based on 14 days of IV antibiotic therapy comprising tobramycin and meropenem with the addition of trimethoprim–sulfamethoxazole (*n* = 4), ceftazidime (*n* = 5) and chloramphenicol (*n* = 2). Antibiotics were also administered via a nebuliser (tobramycin or meropenem) on 13 occasions, generally for 12 weeks [26]. Lastly, Garcia et al. proposed a standardized aggressive protocol comprising a 21-day induction stage (IV + inhaled tobramycin, cetazidime, trimethoprim–sulfamethoxazole) and a 2 months consolidation stage (trimethoprim–sulfamethoxazole, inhaled tobramycin), combined with long-term azithromycin as anti-inflammatory [33]. However, due to the low numbers of patients and the diversity of patient populations and treatment protocols, the best antibiotic strategy is not defined to date [18]. *Burkholderia* species are innately resistant to many antibiotics including some of those used to treat *Pseudomonas aeruginosa* such as aminoglycosides and colistine, and additional resistance may appear during antibiotic therapy, as observed in 2 of our 11 treated patients. Hence, the treatment of colonisations and infections with *Burkholderia* is particularly difficult and based on a small number of molecules mostly used in combination to limit resistance selection. These currently comprise meropenem, ceftazidime, temocillin, cotrimoxazole, ciprofloxacin or cyclines [34]. BCC is usually considered to be resistant to aztreonam, but some strains are susceptible, and Iglesias et al. reported successful eradication of BCC in two patients with bronchiectasis following inhaled aztreonam lysine courses [35]. The latest ceftazidime-avibactam combination therapy seems promising but insufficient clinical experience has been acquired to date [36]. In spite of the natural resistance to aminoglycosides, the use of inhaled tobramycin which allows the delivery of high doses are considered to be of interest in BCC infections [24, 25]. *B. gladioli* is generally slightly more susceptible than BCC, especially to piperacillin-tazobactam and aminoglycosides [17]. The European Committee on Antimicrobial Susceptibility Testing (EUCAST) does not currently advocate *Burkholderia* susceptibility testing as a guideline for antibiotic therapy given the lack of any obvious correlation between in vitro susceptibility and the patient’s clinical course [19]. Conversely, both the Clinical and Laboratory Standards Institute (CLSI) and the antibiogram committee of the French Society for Microbiology provide specific guidelines for a limited number of molecules. In our study, consistency between treatment and antibiotic susceptibility appears to be associated with improved microbiological efficacy, since eradication was achieved in 5/6 cases (83.3%) of antibiogram consistency versus 1/5 cases (20%) of inconsistency.

### Limitations and perspectives

The present study has several limitations, due to the low number of patients, the diversity of species involved as well as of therapeutic approaches, leading to insufficient statistical power. Thus, the positive impact of successful eradication on lung function did not reach statistical significance. Though our results suggest that the antibiogram may be of interest in order to guide therapeutic choices on a case-by-case basis, establishing the relevance of in vitro testing suffers from poor and controversial definition of clinical breakpoints, and from insufficient data. Therefore, we plan to launch a nation-wide retrospective study, which is expected to provide further information and improve statistical relevance.

Moreover, the lack of standardisation in retrospective studies is a potential source of bias. Due to the heterogeneity of the population of CF patients, clinical trials assessing different treatment protocols will probably be required to determine management algorithms considering all clinical and microbiological situations (extent of pulmonary impairment, *Burkholderia* species, co-pathogens). Based on our results and the literature, testing a triple therapy comprising an IV beta-lactam (ceftazidime or meropenem), an oral or IV fluoroquinolone (ciprofloxacin or levofloxacin) plus an inhaled aminoglycoside (tobramycin), and checking for consistency between this protocol and the resistance profile of the isolated strain should be considered when designing such trials. In case of *B. gladioli* colonisation, piperacillin-tazobactam or meropenem could be preferred to ceftazidime owing to their higher in vitro activity [17, 36]. In children, trimethoprim-sulfamethoxazole represents an alternative to quinolones. IV/oral treatments could be administered during 2 to 3 weeks whereas longer durations (8 to 12 weeks) could be proposed for inhaled treatments [26, 33]. Lastly, given the specific pharmacodynamic profile of CF subjects, antibiotic assays should be encouraged for the purpose of dose optimisation. Given the low incidence of the *Burkholderia* infection in CF patients, only large-scale multicentre studies can shed light on this issue.

## Conclusion

The present retrospective pilot study in five French CF Centres highlighted the heterogeneity of therapeutic management of initial infections with *Burkholderia* species. Though not statistically significant, treatment efficacy tended to be related to a better lung function at PC, and to consistency with in vitro antibiotic activity. A nation-wide study is required to evaluate the relevance of these preliminary results. Based on our observations, we suggest the implementation and evaluation of a triple eradication therapy comprising an IV beta-lactam: meropenem, or ceftazidime (BCC), or piperacillin-tazobactam (*B. gladioli*), an oral or IV fluoroquinolone (ciprofloxacin) and an inhaled aminoglycoside (tobramycin). Owing to potential spontaneous clearance, a follow-up sputum culture can be discussed prior to introducing treatment in milder cases. Large-scale studies are required to standardise *Burkholderia* PC treatment in CF patients.

## Data Availability

The datasets used and/or analysed during the current study are available from the corresponding author on reasonable request.

## Abbreviations

ABPA: Allergic broncho-pulmonary aspergillosis
BCC: *Burkholderia cepacia* complex
BMI: Body mass index
CF: Cystic fibrosis
FEV1: Forced expiratory volume in one second
FVC: Forced vital capacity
IV: Intravenous
OBC: Observatoire *Burkholderia cepacia*
PC: Primary colonisation
